# High neural noise in autism: A hypothesis currently at the nexus of explanatory power

**DOI:** 10.1016/j.heliyon.2024.e40842

**Published:** 2024-11-29

**Authors:** Pratik Raul, Elise Rowe, Jeroen J.A. van Boxtel

**Affiliations:** a Discipline of Psychology, Faculty of Health, University of Canberra, Canberra, Australia; b Melbourne School of Psychological Sciences, University of Melbourne, Melbourne, Australia; c Turner Institute for Brain and Mental Health, School of Psychological Sciences, Monash University, Melbourne, Australia

**Keywords:** Autism, Neural noise, Stochastic resonance, Enhanced performance, Reduced performance

## Abstract

Autism is a neurodevelopmental difference associated with specific autistic experiences and characteristics. Early models such as Weak Central Coherence and Enhanced Perceptual Functioning have tried to capture complex autistic behaviours in a single framework, however, these models lacked a neurobiological explanation. Conversely, current neurobiological theories of autism at the cellular and network levels suggest excitation/inhibition imbalances lead to high neural noise (or, a ‘noisy brain’) but lack a thorough explanation of how autistic behaviours occur. Critically, around 15 years ago, it was proposed that high neural noise in autism produced a stochastic resonance (SR) effect, a phenomenon where optimal amounts of noise improve signal quality. High neural noise can thus capture both the enhanced (through SR) and reduced performance observed in autistic individuals during certain tasks. Here, we provide a review and perspective that positions the “high neural noise” hypothesis in autism as best placed to provide research direction and impetus. Emphasis is placed on evidence for SR in autism, as this promising prediction has not yet been reviewed in the literature. Using this updated approach towards autism, we can explain a spectrum of autistic experiences all through a neurobiological lens. This approach can further aid in developing specific support or services for autism.

## List of abbreviations:

DSM: The Diagnostic and Statistical Manual of Mental Disorders
SR: Stochastic resonance
WCC: Weak Central Coherence
EPF: Enhanced Perceptual Functioning
E/I: Excitation/Inhibition
GABA: Gamma-aminobutyric acid
EEG: Electroencephalogram
HNSR: High Noise- SR or High Noise- Stochastic resonance
AQ: Autism Quotient.

## 1 Introduction and background

Autism is a neurodevelopmental difference associated with specific experiences and characteristics. Most studies on autism revolve around explaining *reduced* performances in certain tasks when compared to allistic (or non-autistic) individuals. Such approaches tend to emphasise behavioural and cognitive issues, potentially driven by clinical manuals such as The Diagnostic and Statistical Manual of Mental Disorders (DSM-V-Text Revision), which describes autism as a developmental disorder^1^ characterised by difficulties in social communication, hyper/hypo-sensory sensitivities, and repetitive behaviours [2]. Common cognitive-perceptual issues in autistic individuals^2^ include general reductions in working memory abilities [4], atypical face processing from an early age [5] and issues with pragmatic language which may consequently explain difficulties in social communication [6]. Although enhanced abilities have been reported in autism [[7], [8], [9], [10], [11]], less research has focused on these enhancements. Importantly, a current neurobiological model that captures both enhanced and reduced performance in a single framework is lacking.

Specific autistic experiences and characteristics have been explained by various underlying mechanisms. Some research approaches autism at a behavioural level, and conceptual models, which we categorise here as high-level explanations. Other research approaches it through genetic and network-level causes, which we view as low-level explanations. Lastly, other approaches combine computational and biological-level explanations, which we will categorise as mid-level explanations. We will discuss these approaches in this order, to show the strengths and limitations of these approaches, and why the mid-level explanation currently is the most important approach towards explaining specific autistic behaviours.

Of the mid-level models, we will emphasise the high neural noise hypothesis and argue that this mid-level hypothesis currently has the right balance between computational and predictive power, as well as biological relevance. We focus on a phenomenon called **stochastic resonance (SR)**, or stochastic facilitation [12], as this phenomenon has the potential to explain a variety of autistic experiences and characteristics. SR was initially proposed about 15 years ago by Simmons and colleagues [13] to explain enhanced perception in autism, in light of the general findings of increased noise. However, the hypothesis was, until recently never explicitly tested.

## 2 High and low-level theories of autism

Some of the early models of autism were high-level, conceptual models of autism, such as the Weak Central Coherence (WCC) and the Enhanced Perceptual Functioning (EPF) theory, which focused on explaining the underlying reduced or enhanced mechanisms of observable autistic behaviours. The WCC theory suggests that these altered abilities, and the non-social features of autism, arise from a tendency to process local versus global information [14], resulting from difficulties in constructing the global/contextual aspects of stimuli. The EPF theory suggests that autism is characterised by heightened responses to sensory stimulation at an early stage, which leads to an attentional focus on low-level local/sensory information, at the expense of global information [15,16] (However, also see Ref. [17]). Although the WCC and EPF have been very influential theories, these models lack a neurobiological explanation and predictive power, partly due to the absence of a computational underpinning. As autism is defined as a neurodevelopmental difference, the lack of a neurobiological explanation presents as a critical limitation that needs to be addressed and incorporated for a more comprehensive understanding and better specific support or services for autistic individuals.

On the other end, low-level approaches to explain autism such as the genetic model, aim to explain the aetiology of autism. For example, a systematic review of genetic links to autism showed that some common gene variants such as *SLC25A12, OXTR, RELN, JARID2, CYFIP1, PTCHD1,* and *KDM5B* are associated with an increased likelihood of being autistic [18]. Another meta-analysis showed that mutations of the *SHANK* genes were associated with cognitive difficulties in autism [19]. Moreover, genetic mutations may also contribute to language difficulties in autism [20]. Interestingly, the high co-occurrence of epilepsy in autism can also be explained by genetic mutations. While around two to three per cent of neurotypical children have epilepsy, this percentage increases to 30 % in autistic individuals [21]. Recently, at least 205 common genes (out of 1707 unique genes) were identified that were involved in both epilepsy and autism [22]. Thus, a mutation in these “common genes” may contribute to the development of both autism and epilepsy, potentially explaining the high co-occurrence.

Furthermore, genetic mutations have been associated with network-level changes in autism, such as excitation (E) and inhibition (I) imbalances in the brain [23]. E/I balance is important for optimal neural signal formation, synchrony, and transmission, which supports information processes that drive behaviours [24]. In autism, support for E/I imbalance comes from experimental studies showing dysfunction of gamma-aminobutyric acid or GABA (an inhibitory neurotransmitter) signalling [25,26], resulting in increased excitation and/or decreased inhibition [23,27]. E/I imbalance as a low-level hypothesis can explain anatomical, behavioural, intellectual, language, motor, and social issues in autism [[28], [29], [30], [31], [32], [33], [34]]. It also captures the high co-occurrence of epilepsy in autism [35], as an E/I imbalance also underlies epilepsy [36].

In this context, low-level genetic and network-level models can be combined to explain autism. For instance, many different genetic mutations disrupt synaptic function (i.e., synaptic plasticity, neuronal excitability, and neuronal connectivity), which leads to E/I imbalances resulting in specific autistic experiences and characteristics mentioned earlier [[28], [29], [30], [31], [32], [33], [34]]. Therefore, models like E/I imbalance can provide clearer guidance on what gene mutations to target in future research directions.

A recent review also attempted to bridge computational, algorithmic, and neural instantiation levels of explanation, to explain autism [37]. The authors argued that autism can mainly be explained as a condition of causal inference (at the computational level), a process of ascribing observations to hidden causes [38]. The authors proposed that causal inference relies on other algorithmic processes that are affected in autism due to E/I imbalances (neural implementational level). Whilst this bridging theory focused on E/I imbalances for the neural implementational level, the authors acknowledged that direct experimental evidence for certain aspects of their proposal is still limited at present time [37]. Nevertheless, the causal inference theory may interact with other autism models which is explored in a later section.

There are a few limitations to the low-level approaches. For instance, genetic models cannot entirely capture the complex autistic experiences that are often observed in clinical settings [39], and genetic mutations tend to explain only a small percentage of autism occurrence [40]. Furthermore, as genetic studies are often data-driven as opposed to theory-based, previous findings do not provide much guidance on research directions, without incorporating higher-level explanations. Indeed, in the previous paragraph, the genetic findings were cast in E/I imbalance to gain insight. However, the E/I imbalance model is challenging to implement in psychophysical settings. It is also not rich enough to capture all the neural circuit changes (or joint alterations in neural firing rates) observed in neurodevelopmental conditions [41]. Lastly, the E/I imbalance approach cannot accurately capture enhanced and decreased performance in cognitive tasks.

Therefore, due to the limitations of both high and low-level models, we believe that a mid-level explanation would be a better-suited approach.

## 3 Mid-level explanation of autism: High neural noise models for autism

Building upon the neural aetiology of autism is the ‘high neural noise’ theory which removes some of the precise biological focus of previous theories but gains in formal computational approaches. For example, the high neural noise model fits with well-established psychophysical theories such as Signal Detection Theory [42], thus providing a solid theoretical basis for data analysis and prediction. The theory proposes that whilst an E/I imbalance is associated with higher neural noise, the E/I imbalance itself is not the ‘neural noise’ as there are many other sources of neural noise in autism, such as the dysfunction of neuromodulatory transmitters like dopamine in the autistic brain [43,44], and other abnormalities that occur at lower levels [45]. Thus, an E/I imbalance in the autistic brain may be one way in which neuronal function is made more unreliable, leading to more “noise” within the brain. The high noise explanation, therefore, encompasses the E/I imbalance explanation (along with its computational “sister model” such as divisive normalisation), while also explaining the specific autistic characteristics and atypical brain development [23].

An additional advantage of the high neural noise explanation is that neural noise (i.e., variability) can be directly measured using neurophysiological measures (such as electroencephalography or EEG), while there is no such equivalent for other models. Indeed, EEG recordings have shown high intra-participant neural variability (i.e., trial-by-trial variability) for autism when compared to allistic groups [46,47]. However, studies where no difference between both groups was found, have also been reported in somatosensory and visual tasks [48].

This mid-level explanation can also explain some of the anatomical features of autism, such as atypical functional connectivity, and less developed white matter tracts in autistic brains [45]. For instance, the presence of high amounts of local independent neural noise in one brain area could reduce the functional connectivity with other areas, due to uncorrelated noise amongst brain regions, and if present during early development, the reduced correlation in activity across brain regions may result in atypical anatomical connectivity [45]. An example of atypical connectivity would be the underconnectivity of brain regions which is well documented in the autism literature [49]. The high neural noise theory may therefore be intricately linked not just to the theory of E/I imbalance, but also to the hypothesis of over/underconnectivity in the autistic brain. Additionally, atypical anatomical connectivity can disrupt neural plasticity, contributing to high neural noise in autism [50]. Although a ‘low neuronal noise’ hypothesis for autism has also been proposed in the literature [[51], [52], [53]], some of these studies have been challenged and reinterpreted as evidence for high neural noise [54].

Given the evidence, we believe that the high noise model represents a strong potential explanation for autism. In the sections below, we will summarise the explanatory power of the high noise accounts of autism, focussing on how high neural noise can explain specific autistic experiences and characteristics, enhanced abilities, and its co-occurrence with other conditions such as synaesthesia and epilepsy. Finally, we will conclude with an expanded focus on the ability of the high noise account to explain enhanced abilities through SR.

### 3.1 High neural noise explains specific autistic experiences and characteristics

High neural noise may underpin some behaviours in autism which currently lack a comprehensive explanation [23]. For instance, excessive neural noise is hypothesised to produce unreliable and unpredictable perceptions and representations of the environment [45]. This is especially true for the processing of human emotions and behaviours which are considered complex [55]. Therefore, at an early age, an autistic infant may engage in behaviours that produce predictable responses (such as repetitive behaviours) and avoid unpredictable responses such as social engagements [45]. Thus, high neural noise accounts for social communication issues often associated with autism.

High neural noise can also explain the atypical perceptual effects observed in autism. For example, an experiment showed that autistic individuals have increased neural noise and a reduced ability to filter out external noise [56]. The neural noise estimates also correlated with the severity of specific autistic experiences and characteristics [56]. Interestingly, allistic individuals with high autistic traits also show higher levels of neural noise [57,58]. High neural noise also accounts for atypical low-level processing, such as high visual motion-coherence thresholds [59] (however, see Ref. [60]). Taken together, these results suggest that the high neural noise hypothesis can describe specific autistic experiences and characteristics observed in behavioural data in autism.

### 3.2 High neural noise explains enhanced abilities in autism

**Fig. 1:**
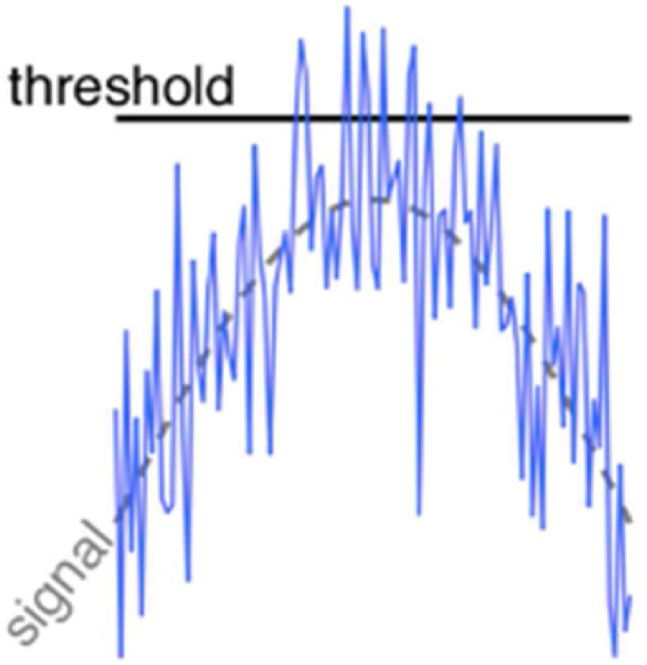
Image illustrating the workings of SR. The signal without noise is not detectable as it is below the detection threshold of a system. However, when an optimal amount of noise is added, the variability offered by the noise helps boost the signal beyond the threshold.

Increased neural noise in autism can also have beneficial effects and can describe enhanced performance in autism through a phenomenon called SR [61,62]. SR is a phenomenon in which a weak signal is amplified above the detection threshold by the addition of an appropriate intermediate level of noise (Fig. 1) [[63], [64], [65]]. Noise levels that are too low would not boost the activity sufficiently to pass threshold, while noise levels that are too high would swamp the signal. Both these latter cases lead to lower performance than intermediate noise levels. While we review the evidence for SR in autism more extensively later, we give a brief primer here.

Bertone and colleagues [66] showed a small enhancement in a first-order (simple) contrast detection-in-noise task in autism when compared to an allistic group, whilst the performance of the autistic group declined in a second-order (complex) task. This pattern of enhanced and reduced performance in autism could be explained by high neural noise and the effects of SR [13]. Higher neural noise in autistic individuals might improve detection performance during simple tasks by pushing a sub-threshold signal above the detection threshold (in an SR-like phenomenon). However, when a task already involves a lot of noise (or is complex) then the high neural noise of the autistic group reduces performance due to decreased signal-to-noise ratios. These results suggest that the high neural noise hypothesis and the effects of SR can explain some of the enhanced performances observed in autism.

### 3.3 High neural noise explains autism co-occurring with epilepsy and synaesthesia

Increased neural noise has been linked to two neurological conditions, epilepsy and synaesthesia which both display increased co-occurrence with autism, suggesting that high neural noise and SR in autism can be linked to other common co-occurring conditions.

**Epilepsy.** As discussed earlier, an autistic brain with high levels of neural noise has an increased likelihood of developing seizures [67] and the high neural noise hypothesis can be used to explain seizure activity. Theoretical and *in vitro* studies suggest that through increased random synaptic noise, the epileptic focus is driven beyond the seizure threshold without any direct manipulation of neurons within the focus itself [68]. Therefore, a brain with high neural noise (like in autism) is susceptible to seizures.

**Synaesthesia.** Synaesthesia is a sensory experience that can be explained as the mixing of senses whereby specific sensory stimuli produce additional unusual perceptual experiences [69]. For example, individuals might experience words or music as colours or see achromatic letters as being coloured, and in rare cases, even taste words [70]. Experimental evidence indicates that synaesthetes show higher autistic traits, atypical sensory sensitivity, decreased global motion processing, and enhanced local elements processing when compared to non-synaesthetes [69] consistent with results found in autism. Interestingly, Lalwani and Brang [71] proposed a model based on excessive neural noise to explain synaesthesia. The authors proposed an SR model where increased neuronal noise would amplify the signals coming through pre-existing multisensory pathways. SR then results in the co-activation of concurrent (for example, colour) with inducer representations (for example, letter) which over a period develops into more stable synaesthetic experiences.

## 4 Summarising the advantages of the high neural noise explanation of autism

**Fig. 2:**
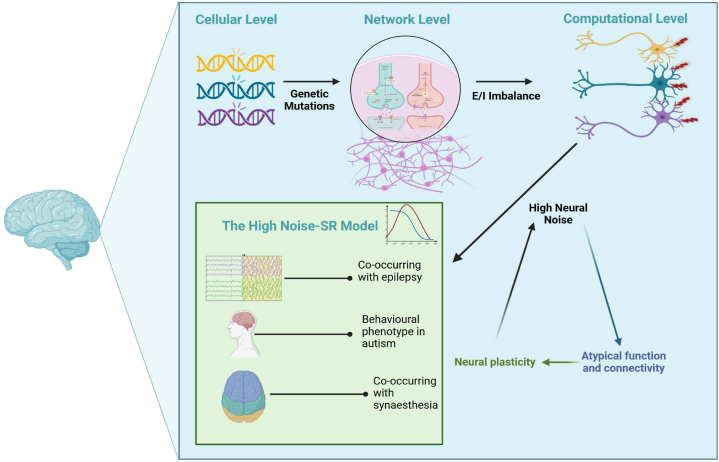
Figure illustrating the summary of the discussed literature.

We examined several levels of explanation for the observed specific phenotypical and neurobiological characteristics of autism. A summary of the above-discussed literature is illustrated in Fig. 2. These levels of explanation are, of course, not mutually exclusive, and different levels of explanation have their advantages [37]. However, we think that currently, the most comprehensive approach that explains most autistic features is the high neural noise theory. Yet, when addressing specific research questions, it may be beneficial to focus more on other linked explanations such as differences in connectivity, or E/I balance theories.

Predictive coding theory accounts of autism also provide a strong theory that encompasses many autistic characteristics and experiences in a single framework [72,73]. While we acknowledge the strength of the predictive coding accounts of autism and the possibility that it may subsume the high-noise explanation (either as increased noise on likelihoods or priors, or both), we favour the high-noise account for its unambiguity and its simplicity. Also, the high neural noise approach does not suffer the same concerns that predictive coding has faced, namely, that it is too complex and too difficult to falsify [74,75].

In summary, the promising ‘high neural noise’ theory proposes that genetic issues disrupt synaptic regulation which leads to E/I imbalances that can result in a brain with high noise in autism. This hypothesis is better at supplying predictions, explains increased and reduced sensitivities in autism, and has the power to explain atypical brain structure and function, social issues, and reduced and enhanced abilities in attentional and perceptual tasks observed in autism, as well as the reason for co-occurrences with epilepsy and synaesthesia. We note that many of these proposed explanations also depend on SR. However, despite the potential of this model to explain various autistic behaviours from a neurobiological perspective, there has been no concerted effort to investigate the link between SR, high neural noise, and the specific autistic behavioural phenotype. In the remainder of this article, we will review evidence in favour of the SR theory of autism.

## 5 SR and autism: The high noise-SR (HNSR) model

**Fig. 3:**
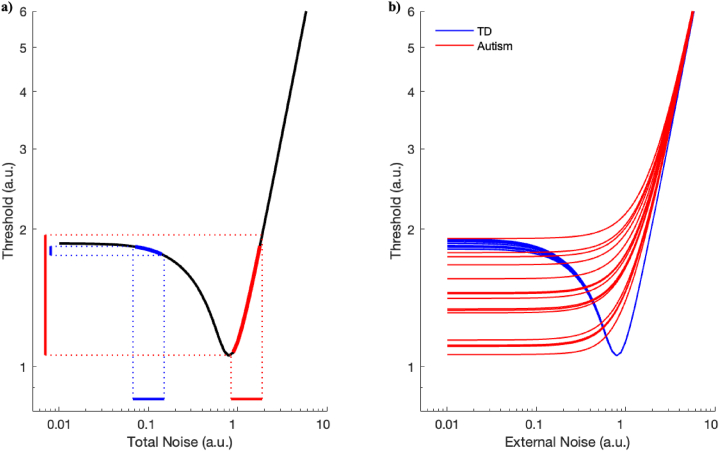
Visual depiction of the HNSR model. Both figures show threshold versus noise SR plots. Lower thresholds indicate better/improved performance. Both axes are in arbitrary units (a.u.).

In SR, adding moderate amounts of noise will improve detection (i.e., reduce the threshold or increase sensitivity), unless the noise is too high, in which case detection performance is impeded. Such a scenario is depicted in Fig. 3a, where the x-axis denotes total noise, and the y-axis represents the detection threshold (where lower thresholds indicate better performance). The black line illustrates the threshold versus the total noise curve, depicting the standard SR improvement within a particular intermediate level of total noise. We have previously presented computational approaches to model both accuracy [61] and d’ [76], in this context. The latter of which is used to create Fig. 3.

Fig. 3a shows performance against overall levels of noise. This is a combination of noise in the proximal stimulus (external noise) and noise from within the neural system (neural noise). Now suppose that there are two groups of people, one with low levels of neural noise (e.g., allistic individuals; blue in Fig. 3a and b), and the other with higher levels of neural noise (the autistic group; red in Fig. 3a and b). Their ranges of neural noise are shown as coloured bars on the x-axis. If this noise were the only noise, then the red group (i.e., autism) is already at the optimal position and has better performance than the blue group. Adding any type of noise will only reduce their performance. Alternatively, the blue group can benefit from increased levels of external noise, moving through the dip of the curve (i.e., best performance) before higher levels of noise start to deteriorate performance.

This pattern of results is clearer when plotted against external noise (which is experimentally manipulable) instead of total noise (Fig. 3b). Here, adding similar levels of external noise in both groups leads to two results: 1) at low noise (<1 external noise), the autistic group outperforms the allistic group, and 2) at the ‘optimal’ amount of noise (∼1 external noise), the allistic group shows SR dependent on external noise, but the autistic group does not. These effects occur because the allistic group can benefit from the addition of the external noise via SR while the autistic group cannot (as it was already at the optimal noise level before the addition of external noise). Overall, this explains why some autistic individuals can outperform allistic individuals in certain tasks but then show reduced performance in a more complex variation of the same task [66].

Additionally, when we account for variability in the groups (i.e., equivalent levels of variation in neural noise (in log space) within each group, i.e., the blue and red bars at the bottom of Fig. 3a) and drawing a random sample (see different lines in Fig. 3b), we see that whilst average thresholds are lower in the autistic group (Fig. 3b), the variance is much higher compared to the allistic group which is consistent with the literature [47,77].

The outcomes presented here depend on the specific parameters chosen, but they do show that SR is a possible explanation for the superior performance in autism, despite increased levels of neural noise. Dwyer and colleagues also suggested that the balance of signal-to-noise ratio in autism is complex and context dependent and this is true if SR is used as an explanation for enhanced performances in autism [52]. Also, the larger inter-individual variability for autism would mean that some autistic individuals may not show this superior performance despite high neural noise levels which could also account for inconsistencies in the literature [52].

### 5.1 Supporting evidence for the HNSR model

**Fig. 4:**
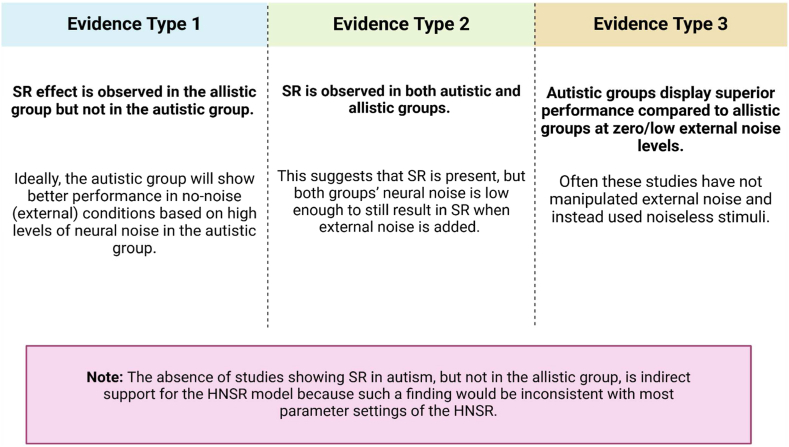
The figure illustrates the three types of evidence that can support the HNSR model for autism.

We review three types of evidence (Fig. 4) that would provide direct or indirect support for the HNSR model.

#### 5.1.1 Evidence type 1: SR observed for allistic but not in autistic groups

Perhaps the strongest empirical support for the HNSR model comes from the findings of Zaidel and colleagues [78]. Here, autistic adolescents and age-matched allistic participants performed self-motion perception tasks. The authors showed that global and multisensory integration remained intact in autism, however, autistic individuals had difficulties from heightened noise sensitivity which the authors attributed to higher reliance on incoming sensory signals and less use of prior knowledge of the world [72].

**Fig. 5:**
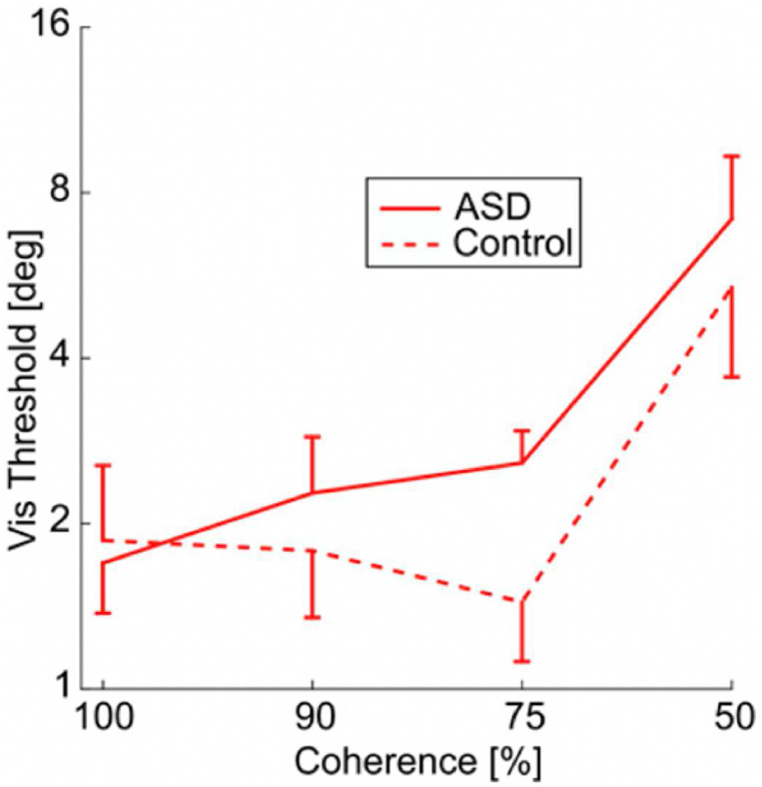
Figure shows responses to visual noise in the autistic and allistic group [78]. Lower thresholds indicate better performance. Red lines represent the log-scale mean visual thresholds, as a function of visual motion coherence. Image used with permission.

Fig. 5 shows the effect of increased external noise (i.e., reduced motion coherence) on performance (i.e. threshold) [78]. An SR effect is observed for the allistic group when external visual noise is introduced by decreasing the coherence level to 90 and 75 %. No SR occurs in the autistic group. However, at the zero-noise condition (100 % coherence), the autistic group displayed lower threshold levels (i.e., better performance) compared to the allistic group. Importantly, these results provide empirical support for the model shown in Fig. 3b and suggest that increased neural noise for the autistic group leads to improved performance in the zero-noise condition and an SR effect (due to external noise) only in the allistic group.

Note that the overall results of the Zaidel and colleagues study support the atypical causal inference theory in autism [37]. The causal inference theory and HNSR hypothesis are not mutually exclusive. Causal inference relies on a process of divisive normalisation [37]. Divisive normalisation is a mechanism that is involved in counter-acting neural noise [79], and this mechanism is atypical in autism [37] which could also explain the high neural noise (and therefore, instances of SR) in autism.

Similarly, in a visual detection task performed in our laboratory [61], we showed that participants with higher autistic traits (as measured by their Autism Quotient, AQ [80]) performed better at zero- and low-levels of external noise compared to lower AQ participants. Moreover, performance was reduced at high external noise levels for the higher AQ group. However, in this experiment, the low AQ participants did not show a clear SR effect at intermediate external noise levels. Thus, these findings again partly support our HNSR model.

We also want to acknowledge studies where such advantage in noise-based tasks was not found in autism. For example, in a recent study no differences in mean discrimination thresholds were observed between autistic and allistic individuals at zero or low external visual noise levels in a motion discrimination task [81]. In another study, participants were asked to detect the presence or absence of contours that were embedded among similar Gabor elements [82]. Although the results were noisy overall, there was a presence of SR for the allistic group but not for the autistic group (i.e., the best performance was at any non-zero external noise level for the allistic group). However, there was no advantage for the autistic group at zero noise (i.e., no SR), therefore, only partly supporting the HNSR model.

In a more recent study by Mihaylova and colleagues, the authors investigated the effects of visual noise on reading performance in three neurodevelopmental conditions: autism, attention deficit hyperactivity disorder, and developmental dyslexia [83]. Autistic participants showed an advantage in reading time per letter string for pseudowords containing seven letters when no noise was present (note that in other conditions, the reading times for the autistic group were comparable to those of the allistic group). This finding aligns with the predictions of the HNSR model. When external visual noise was introduced, all groups, including allistic group, experienced a decline in performance. However, the autistic group showed the least decline. Moreover, at the highest noise level, autistic participants still performed better than all other groups. This result does not align with the predictions of the HNSR model, and we are currently unsure how to best explain these findings.

Overall, several recent studies show varying degrees of experimental support for the HNSR model, with the data by Zaidel and colleagues [78] capturing the predicted pattern most accurately. These results indicate that the effects of high neural noise and SR in autism can be captured via experimental studies, underlining the explanatory power of the HNSR model.

#### 5.1.2 Evidence type 2: SR observed for both autistic and allistic groups

A few studies have found evidence for tasks in which SR is present in both autistic and allistic groups. For instance, in a coarse orientation discrimination task with a large stimulus size and low spatial frequency, external noise was introduced through Gaussian pixel noise [56]. Evidence was found for increased levels of neural noise in autism. The results also displayed the characteristic SR pattern for both the autistic and allistic groups, suggesting that while neural noise was higher in the autistic group, it was low enough to allow for external noise-induced SR [56]. To complement these findings, we highlight further results from our group where we compared the effects of SR in a visual identification task in noise in the general population with either high or low AQ [61]. We showed that SR was present for both higher and lower AQ participants. However, here we did not find evidence for enhanced performance (SR due to neural noise) in the high AQ group at zero or low external noise levels. These results support the HNSR model in that when neural noise is low enough, SR can be shown by autistic groups.

#### 5.1.3 Evidence type 3: Enhanced performance for autistic group in other perceptual tasks

As discussed earlier, autistic individuals show small enhancements in first-order (simple) contrast detection tasks in low amounts of noise reportedly due to their neural noise-inducing SR [66]. However, performance is reduced for second-order (complex) tasks as further increases in noise may overwhelm the autistic brain making extracting information difficult. These results align with the HNSR model of the enhancement of some behaviours in autism. Such findings may also explain why autistic individuals show enhanced detection of details but have difficulties with other tasks such as face perception [84], as faces require the processing of more complex information which further increases neural noise. There are various other tasks in which autistic individuals show better performance than allistic individuals. These tasks include visual search tasks [7], embedded figures [85] and block design tasks [86]. These results also align with the SR hypothesis of increased performance when external noise is absent hence, further providing support towards the HNSR model.

Note that, although these results align with the idea that enhanced performance in autism can be found at low external noise conditions, the high-noise explanation would predict that when external noise is introduced, the autistic group's performance would decrease, while the allistic group would show an increase in performance before a subsequent decrease with ever-increasing noise. These experiments will still need to be performed to provide more complete support for the high-noise hypothesis. We do not know yet what type of noise would work best for these tasks.

## 6 Concluding remarks and future perspectives

Fifteen years have passed since the SR hypothesis for autism was first proposed [13]. Despite a lack of studies designed to investigate this idea directly (until recently [61]), the literature has provided clues to support this hypothesis. Here, we reviewed the evidence in favour of the High noise-SR model (HNSR) [13,61] and its potential to explain the specific phenotypical and neurobiological characteristics of autism. By choosing to focus on SR, we acknowledge an inherent bias in this perspective. However, without this emphasis, the evidence risks being overshadowed by studies that report no differences, even when some were observed. We believe the high-noise explanation of autism is at the nexus of explanatory power. Indeed, the HNSR model aligns with current cellular, neurobiological, and computational models of autism and builds upon the high neural noise hypothesis by describing how high neural noise and SR might underlie the complex behaviours associated with autism.

To provide further insight into the validity of the HNSR model, future studies need to consider the possibility of SR. Much of the empirical evidence for SR in the literature is not mentioned in the original articles, as it was not of main interest, or perhaps it was unexpected, and could not be explained. Additionally, if authors find increased performance in autistic individuals using stimuli without noise, the HNSR model can easily be tested with additional experiments where external noise is added to the stimuli.

In conclusion, the HNSR model attempts to capture various autistic behaviours in a single framework. Such models that highlight the superior abilities in autism are important as they can help provide opportunities (where such skills can be utilised) and reduce stigma or discrimination towards autistic individuals across settings. Furthermore, a better understanding of the neural mechanisms underlying the complex behaviours in autism can aid mental health professionals in more accurate diagnosis of this complex neurodevelopmental difference.

## Available of data and materials

Not applicable.

## CRediT authorship contribution statement

**Pratik Raul:** Writing – original draft. **Elise Rowe:** Writing – review & editing. **Jeroen J.A. van Boxtel:** Writing – review & editing, Supervision.

## Human ethics approval and consent to participate

Not applicable.

## Consent for publication

Not applicable.

## Funding

This research was funded by the Australian Government through the 10.13039/501100000923Australian Research Council Discovery Project (project number DP220100406) awarded to Jeroen J.A. van Boxtel.

## Declaration of competing interest

The authors declare that they have no known competing financial interests or personal relationships that could have appeared to influence the work reported in this paper.

## Footnotes

1 Based on recent literature, in this review, we will continue to use autistic-preferred language and discuss autism not as a disorder but as a neurodevelopmental difference (see Ref. [1]).

2 Note that we will be using identity-first language throughout this manuscript as we are aligned with the consensus of using identity-first language as opposed to person-first language (see Ref. [3]).
